# The Influence of Erosive and Abrasive Effects of Interdental Brushing on Bovine Enamel and Dentine In Vitro

**DOI:** 10.3290/j.ohpd.c_2302

**Published:** 2025-10-22

**Authors:** Lynn V. Etter, Andrea Gubler, Florian J. Wegehaupt, Patrick R. Schmidlin

**Affiliations:** a Lynn V. Etter Dental Master’s student, Clinic of Preventive Dentistry, Periodontology and Cariology, Center for Dental Medicine, University of Zurich, Zurich, Switzerland. Performed the experiments as part of a Master’s thesis, wrote the manuscript.; b Andrea Gubler Lab Manager, Clinic of Preventive Dentistry, Periodontology and Cariology, Center for Dental Medicine, University of Zurich, Zurich, Switzerland. Supported and organised the implementation of the experiments, and proofread the manuscript.; c Florian J. Wegehaupt Professor and Head, Division of Preventive Dentistry and Oral Epidemiology, Clinic of Conservative and Preventive Dentistry, Center for Dental Medicine, University of Zurich, Zurich, Switzerland. Proofread the manuscript.; d Patrick R. Schmidlin Professor, Head Division of Periodontology and Peri-implant Diseases, Clinic of Conservative and Preventive Dentistry, Center for Dental Medicine, University of Zurich, Zurich, Switzerland. Study idea, experimental design, supervision, and proofread the manuscript.

**Keywords:** erosion, abrasion, enamel, dentine, wear

## Abstract

**Purpose:**

To investigate the effects on enamel and dentine caused by erosion with hydrochloric acid (HCl) or abrasion with interdental brushes and toothpaste, and a combined protocol using roughness and profilometry measurements as well as scanning electron microscopy images.

**Materials and Methods:**

24 bovine enamel samples and 24 dentine samples were divided into three groups each (groups 1–3: enamel; groups 4–6: dentin; n = 8) and investigated after an abrasion protocol (2 × 10 brushing cycles per day) in groups 1 and 4, a combined protocol (10 brushing cycles, 10 × 2min erosion in HCl, 10 brushing cycles per day) in groups 2 and 5 and an erosion protocol (10 × 2min erosion in HCl) in groups 3 and 6 for 10 days. Profilometry and roughness measurements were registered at baseline, after 5 and after 10 days. Scanning electron microscopy (SEM) images were recorded at baseline and after 10 days.

**Results:**

For enamel, the significantly highest substance loss (mean ± SD) after 5 days was observed in the combined group (10.71 ± 0.41 μm), whereas the second highest substance loss was in the erosion-only group (9.16 ± 0.23 μm). In dentin, the highest substance loss after 5 days occurred in the combined group (7.4 ± 0.83 μm), and the second highest substance loss was presented in the erosion-only group (7.26 ± 0.5 μm). After 10 days, the combined group (18.88 ± 0.56 μm) exhibited the highest substance loss in enamel, showing a statistically significant difference compared with the erosion-only and abrasion-only groups (P <0.001, respectively). In dentin, the highest substance loss was observed after 10 days in the erosion-only group (7.96 ± 0.8 μm), exhibiting a statistically significant difference compared to both the combined (P = 0.022) and abrasion-only groups (P <0.001).

**Conclusion:**

In all groups, with the exception of abrasion-only groups, enamel substance loss occurred. Thus, measures against erosive and abrasive wear, both alone and combined, have to be implemented, such as comprehensive patient education.

Dental hard tissue wear, including erosion and abrasion, is a growing concern in modern dentistry. With changing dietary habits, increased consumption of acidic beverages, and evolving oral hygiene practices, the prevalence of enamel and dentine wear is rising.^28^ Dental erosion, in particular, has been reported in up to 30% of adults and 50% of children, with varying degrees of severity depending on lifestyle and dietary factors.^23^ Abrasion, often associated with improper brushing techniques and abrasive toothpaste formulations, further contributes to structural loss of dental tissues.^38^ When first erosion and then abrasion act successively, the cumulative effect may lead to rapid tissue degradation, increased hypersensitivity, and even functional impairment of the dentition.^45^ Understanding the processes of erosion and abrasion is essential to developing effective preventive and therapeutic strategies in clinical practice.

Dental erosion is defined as the progressive loss of dental hard tissues due to chemical processes that do not involve bacterial activity. It results from exposure to acidic substances that dissolve the hydroxyapatite crystals of enamel and dentine.^40^ The degree and progression of erosion is affected by salivary properties such as flow rate, buffering capacity, pH value and composition.^24^ Erosion can be classified into intrinsic and extrinsic types based on its aetiology. Intrinsic erosion occurs due to hydrochloric acid (HCl) exposure from within the body. Common causes include gastroesophageal reflux disease (GERD), frequent vomiting in eating disorders such as bulimia nervosa, and alcohol abuse.^13^ This form of erosion primarily affects the palatal and occlusal surfaces of teeth.^24^ Extrinsic erosion is caused by acids from external sources, such as acidic food and beverages (eg, citrus fruits), acidic drugs (eg, chewable vitamin C tablets), or environmental exposure (eg, swimming in chlorinated pools).^24^ In these cases, labial surfaces of anterior teeth and buccal and occlusal surfaces of lower posterior teeth are typically affected.^1^ While acidic environments initially cause softened layers, repeated long-time erosive effects lead to substance loss, thus making teeth more susceptible to mechanical forces such as abrasion.^22^

Tooth abrasion is the mechanical wear of dental tissues caused by friction from external forces, primarily associated with oral hygiene practices and dietary habits.^38^ Unlike erosion, which is chemically driven, abrasion results from repetitive mechanical forces that gradually wear down the tooth surface, with several key factors contributing to abrasion.^27^ Toothbrushing-related abrasion is one of the most common causes.^27^ The combination of aggressive brushing techniques, the use of hard-bristled toothbrushes, and highly abrasive toothpastes can significantly accelerate dentine wear.^38^ Studies have shown that horizontal brushing motions, in particular, lead to more pronounced abrasion compared to vertical or circular techniques.^10^ An *in-vitro* study has shown that interdental brushes (IDBs) in combination with toothpaste also caused substance loss on dentine.^35^ Abrasive dental products, especially whitening toothpastes and charcoal-based formulations, can contribute to mechanical wear.^32^ The abrasiveness of a toothpaste is measured by its relative dentine abrasion (RDA) value and relative enamel abrasion (REA) value, with higher values correlating with increased dentine and enamel loss over time.^19^ Unlike erosion, which primarily affects smooth and non-load-bearing surfaces, abrasion is more localised, often occurring on cervical areas of teeth, particularly in patients with vigorous brushing habits.^17,23^ When combined with erosion, abrasion can lead to more extensive tissue loss, further compromising dental structures.^12^

Erosion demineralises the enamel surface, making it softer and more susceptible to mechanical wear. Subsequent abrasion, such as toothbrushing or IDBs, can then remove these weakened layers, thereby accelerating enamel loss.^22^ Dentine, due to its lower mineral content and tubular structure, is even more vulnerable than enamel.^36^ Once erosion breaches the enamel layer and exposes dentine, the progression of wear increases significantly.^21^ The combined effect of erosion and abrasion on dentine can lead to hypersensitivity and reduce structural integrity, emphasising the need for preventive measures such as fluoride applications, low-abrasion toothpastes, and modifications in oral hygiene practices.^21,45^

To the author’s knowledge, the study of Schmidlin et al^35^ is the only one that investigated the effect of IDBs in combination with toothpaste on dentine. The combined effect of erosion and abrasion with IDBs on dentine wear, as well as the effect of IDBs on enamel wear, has not been investigated to date. The present study, therefore, aims to analyse the impact of erosion by HCl, abrasion by IDBs, and their combined effects on bovine enamel and dentine, simulating clinical conditions to better understand the mechanisms of tissue loss. The first null hypothesis was that there is no difference in enamel wear between the erosion-only, abrasion-only, and combined erosion-abrasion groups. The second null hypothesis was that there is no difference in the wear of dentine between the erosion-only, abrasion-only and combined erosion-abrasion groups. The third null hypothesis was that the effects of different treatment protocols (abrasion-only, erosion-only or combined erosion-abrasion) on wear are independent of material (enamel or dentine).

## MATERIALS AND METHODS

### Sample Preparation

The experimental setup is illustrated in Table 1. For the study, 24 enamel and 24 dentine samples were prepared from anterior bovine teeth. The samples were drilled out of bovine enamel and dentine, respectively, using a hollow drill (BFW 40/E, Proxxon, Föhren, Germany) with an inner diameter of 4 mm. The dentine samples were then shortened at the pulp-near dentine side, and the enamel samples were shortened at the dentine-near side to approximately 2 mm thickness with pliers. Both dentine and enamel samples were then embedded in denture acrylic (Paladur, Heraeus Kulzer, Hanau, Germany) using a silicone template of 6 mm diameter with a notch to ensure correct positioning of the sample in measurement devices. To ensure complete polymerisation, the samples were hardened at 40°C and 2 bar for 8 min in a laboratory incubator (Palamat elite, Heraeus Kulzer, Hanau, Germany). Using a milling machine, the samples were milled from the bottom to a suitable height to fit the brushing machine. The excess denture acrylic on top of the enamel and dentine surfaces was removed with a scalpel. To ensure a standardised surface on all the samples, they were polished in an automatic microprocessor controlled grinding machine (Tegramin-30, Struers; Ballerup, Denmark) with a standardised programme for enamel (1200 Grit, 5 N, 15 s; then 2000 Grit, 5 N, 20 s; then 4000 Grit, 5 N, 30 s) and dentine (2000 grit, 5N, 20s; then 4000 Grit, 5N, 30 s) under constant water cooling. The thickness of the samples was reduced to 2.95 ± 0.03 mm with a milling machine. Finally, a groove was made in the denture acrylic left and right of the dentine, respectively, enamel, as close to the tooth surface as possible, using a sharp needle in a custom-made device. The enamel and dentine samples were then divided into three groups each (n = 8), labelled with numbers and stored in tap water. Four days prior to the start of the study, the samples were transferred to artificial saliva and stored at 37°C. The artificial saliva was prepared according to the recipe of Klimek et al.^25^

### Brushing and Erosion Procedure

**Fig 1:**
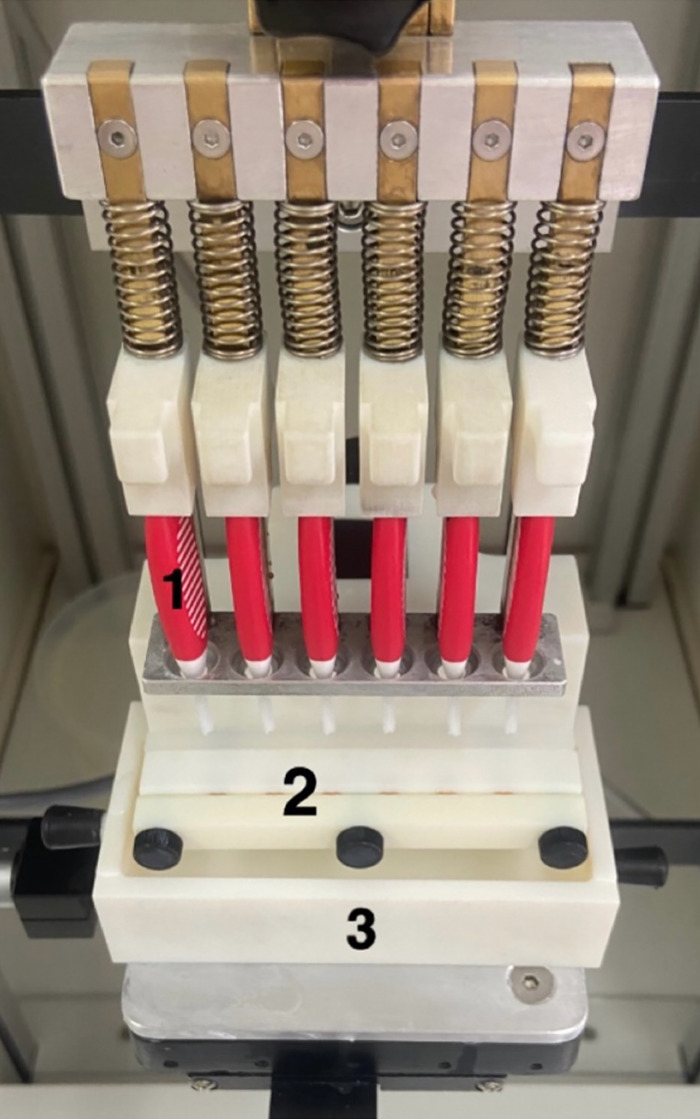
Brushing machine for IDBs (1) with holder for samples (2) and container with toothpaste slurry (3).

Brushing of the samples was performed using a modified, custom-made brushing machine for IDBs (Fig 1, Department of Conservative and Preventive Dentistry, Center for Dental Medicine, University of Zurich, Switzerland).^35^ Six samples were placed into a holder and dipped into the toothpaste slurry, which consisted of one part of Colgate Total Original toothpaste (Colgate-Palmolive; Poland; Lot No. L3251PL1174/L3251PL1175) by weight and 2 parts of artificial saliva by weight. The toothpaste slurry was exchanged each day. At the top of the machine, 6 IDBs (Elmex red, Colgate-Palmolive; Poland; size 2, 0.5 mm, Lot no. 08718951387096), which were numbered for each sample and exchanged after 5 days of use, were inserted. The samples were then treated for 10 cycles, ie, 20 brush strokes twice a day. The samples were removed from the machine, rinsed with deionised water for 30 s, and stored in artificial saliva at 37°C.

**Fig 2:**
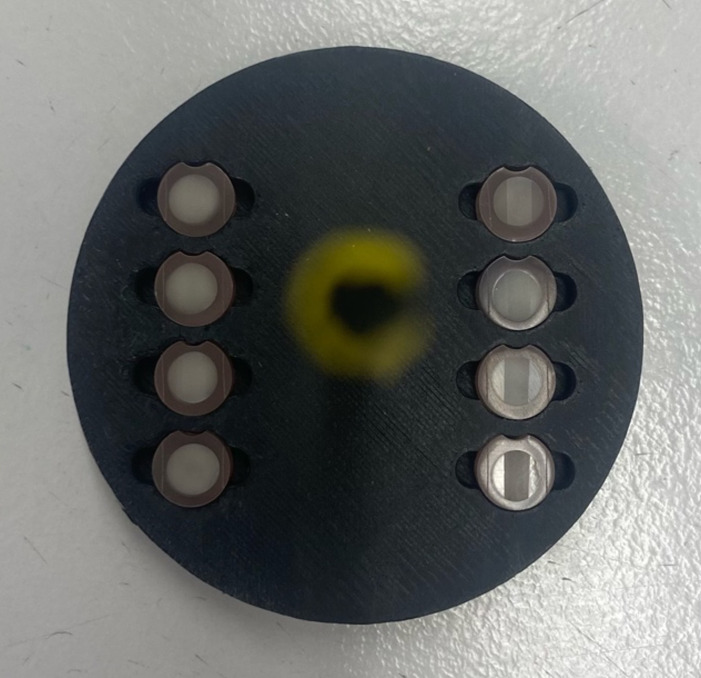
3D printed sample holder for erosion.

For the erosion procedure, the samples were placed in a holder made with a 3D printer per group and taped on both sides with adhesive tape (Scotch Crystal, 3M; Saint Paul, USA) to create a border (Fig 2). For each cycle, the samples were put in hydrochloric acid (pH 2.3) for 2 min. The hydrochloric acid used in the study consisted of 5.02 ml of hydrochloric acid with a concentration of 1 mol/L (Honeywell/Fluka; Charlotte, USA; Lot No. L3000), which was diluted with deionised water to achieve a final volume of 1000 ml and a pH value of 2.3. Subsequently, the samples were rinsed with deionised water for 30 s to stop the erosive attack and placed back in artificial saliva for 20 min. Each day, 10 such cycles were performed. Samples were stored in artificial saliva at 37°C overnight.

### Measurement Methods

Profilometry and roughness measurements were conducted at baseline, after 5 days and after 10 days. During all measurements, samples were kept wet in artificial saliva to avoid dehydration. Due to the notch in the acrylic resin, samples could be measured in the exact same position in all measurements.

The determination of substance loss was performed with a contact profilometer (MFW-250/Perthometer S2, Mahr; Göttingen, Germany). Five parallel lines orthogonal to the grooves that were made for reference were measured with a needle. The lines were 4.8 mm long and had a distance of 250 μm in between. The profiles of the baseline measurements and measurements after 5 and 10 days were then superimposed using the grooves in the denture acrylic to calculate the substance loss.

The roughness was measured using a roughness depth gauge (Form Talysurf Series 2, Taylor Hobson Precision; Leicester, England) to quantify the surface condition of the sample. Using an inductive probe with a tip diameter of 4 μm, three measurements of 1 mm length each were taken per sample at a measuring speed of 0.5 mm/s. One measurement was taken in the middle of the sample, and the other two measurements were taken 1 mm to the left and right of the first measurement.

Scanning electron microscopy (SEM, Zeiss GeminiSEM 450, Zeiss; Oberkochen, Germany) images were taken for baseline samples and samples after 10 days at a magnification of 2000×. Samples after 5 days could not be evaluated due to the fact that there were no samples left. Before taking SEM images, samples were dehydrated after the experiment. Initial dehydration was performed with 50% ethanol. Samples were then left in an ultrasonic bath for 15 min and rinsed with 50% ethanol. Then the standardised dehydration procedure was performed with an ascending ethanol series. With a carbon pad, the samples were fixed to SEM carriers and were sputtered with a 10 nm-thick layer of gold. The samples were always placed in the same position in the SEM, notch downwards, so that the brushing direction is visible vertically. The SEM was operated with a voltage of 10 kV.

### Statistical Analysis

The data (surface loss and surface roughness) were coded and documented using Excel (version 16.70, Microsoft, Redmond, Washington, USA) and statistically analysed using DATAtab Team (2022) (DATAtab: Online Statistics Calculator. DATAtab, e.U. Graz, Austria, URL https://datatab.net). Descriptive statistics were used to describe means, medians, standard deviations, and interquartile ranges (IQR). Statistical analysis was performed separately for dentine and enamel. The distribution of the values was depicted as box plots. Normality was investigated using Kolmogorov–Smirnov and Shapiro–Wilk tests. Since data for surface roughness measurements were not normally distributed, non-parametric Kruskal-Wallis and Mann–Whitney tests were used to assess significant differences between groups. A Bonferroni correction was used for multiple comparisons. Data for surface loss were normally distributed; therefore, results between the treatment groups were analysed using a simple analysis of variance (ANOVA), and Fisher’s least significant difference test was applied. A significance level of P <0.05 was defined for all statistical tests.

### Ethics

The enamel and dentine samples originate from domestic cattle. Generally, bovine teeth are slaughter by-products and thus disposed of. There were therefore no concerns from either an ethical or animal welfare perspective.

### RESULTS

#### Substance loss

**Fig 3:**
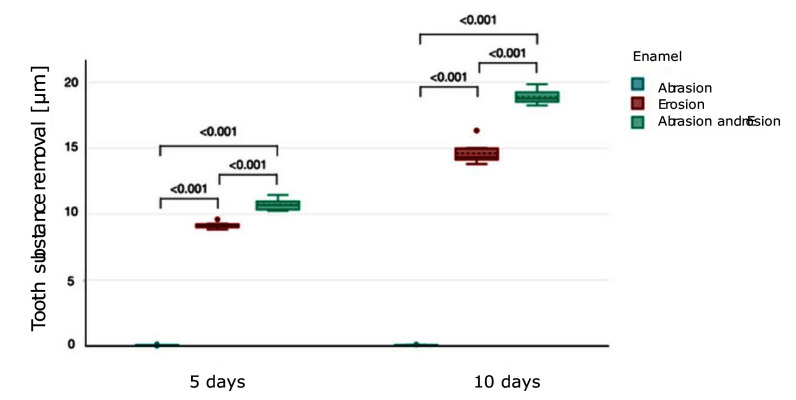
Boxplot showing tooth substance removal (μm) (mean ± SD) in enamel under different conditions over 5 and 10 days. Brackets between the plots represent the P value.

The enamel substance (mean ± SD) loss for each group is shown in Figure 3. After 5 days, enamel subjected to abrasion alone exhibited minimal substance loss (0.06 ± 0.03 μm), whereas erosion led to a significantly higher substance loss (9.16 ± 0.23 μm) (P <0.001). The combination of abrasion and erosion caused the most severe enamel loss (10.71 ± 0.41 μm), which was statistically significantly higher than both individual effects in erosion- and abrasion-only groups (P <0.001). After 10 days, the combined group exhibits the highest enamel loss (18.88 ± 0.56 μm), showing a statistically significant difference vs erosion-only and abrasion-only groups (P <0.001).

**Fig 4:**
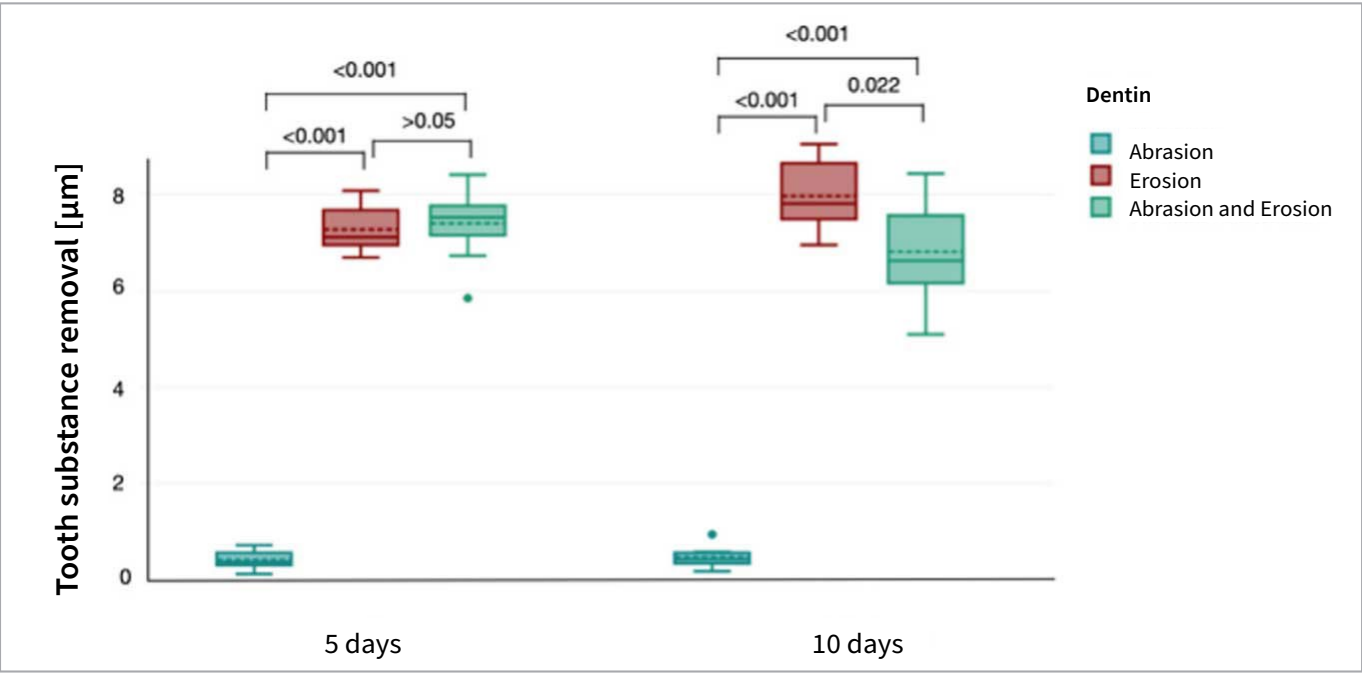
Boxplot showing tooth substance removal (μm) (mean ± SD) in dentine under different conditions over 5 and 10 days. Brackets between the plots represent the P value.

The dentine substance (mean ± SD) loss results are depicted in Figure 4. After 5 days, both erosion only (7.26 ± 0.5 μm) and the combined group (7.4 ± 0.83 μm) caused significantly higher substance loss compared to abrasion alone (0.43 ± 0.21 μm) (P <0.001), whereas the combined group did not significantly differ from the erosion-only group (P >0.05). After 10 days, substance loss in the erosion-only group (7.96 ± 0.8 μm) was the highest of all groups and resulted in a significantly higher substance loss than in the abrasion-only group (0.48 ± 0.23 μm). There was a statistically significant difference between the erosion-only group and the combined group (6.81 ± 1.09 μm) after 10 days (P = 0.022).

#### Roughness

**Fig 5:**
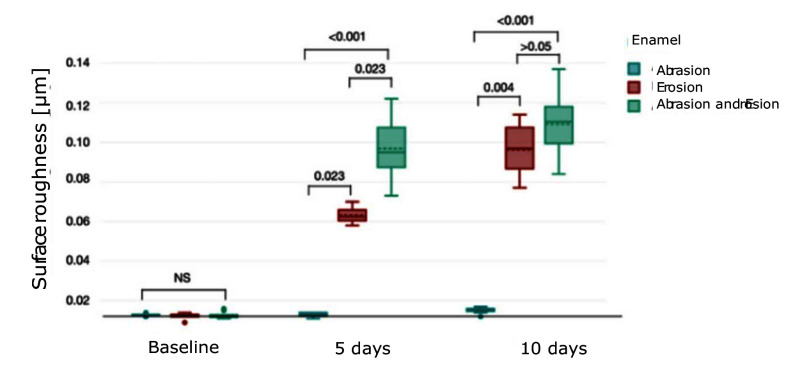
Boxplot showing surface roughness (μm) (median ± IQR) in enamel under different conditions over 5 and 10 days. Brackets between the plots represent the P value.

**Fig 6:**
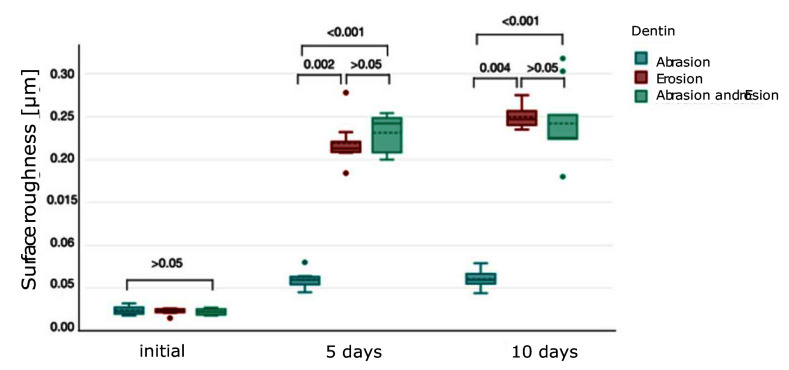
Boxplot showing surface roughness (μm) (median ± IQR) in dentine under different conditions over 5 and 10 days. Brackets between the plots represent the P value.

The surface roughness (median ± IQR) of enamel is depicted in Figure 5. Initially, there were no significant differences among the groups (P >0.05, respectively). After 5 days, the erosion-only group (0.06 ± 0.01 μm) statistically significantly differed from the abrasion-only (0.01 ± 0 μm) and combined group (0.1 ± 0.02 μm) (P = 0.023 for both). There was also a statistically significant difference between the abrasion-only and combined group in enamel after 5 days (P <0.001). After 10 days, enamel subjected to combined abrasion and erosion (0.11 ± 0.02 μm) exhibited significantly increased roughness compared to abrasion alone (0.02 ± 0.01 μm) (P <0.001). The erosion-only group (0.1 ± 0.02 μm) had a statistically significant difference vs the abrasion-only group (P = 0.004), but not vs the combined group (P >0.05). Surface roughness data (median ± IQR) of dentine are presented in Figure 6 and follow a similar trend. Initially, there were no significant differences among the groups (P >0.05, respectively). After 5 days, erosion alone (0.21 ± 0.01 μm) (P = 0.002) and combined abrasion and erosion (0.24 ± 0.04 μm) (P <0.001) resulted in significantly higher roughness compared to abrasion alone (0.06 ± 0.01 μm). After 10 days, the erosion-only group (0.25 ± 0.02 μm) showed the highest roughness, whereas the combined group (0.23 ± 0.03 μm) also expressed a relatively high roughness. There was no statistically significant difference in roughness between the erosion group and the combined group after 5 and 10 days (P >0.05).

#### Scanning electron microscopy (SEM)

**Fig 7:**
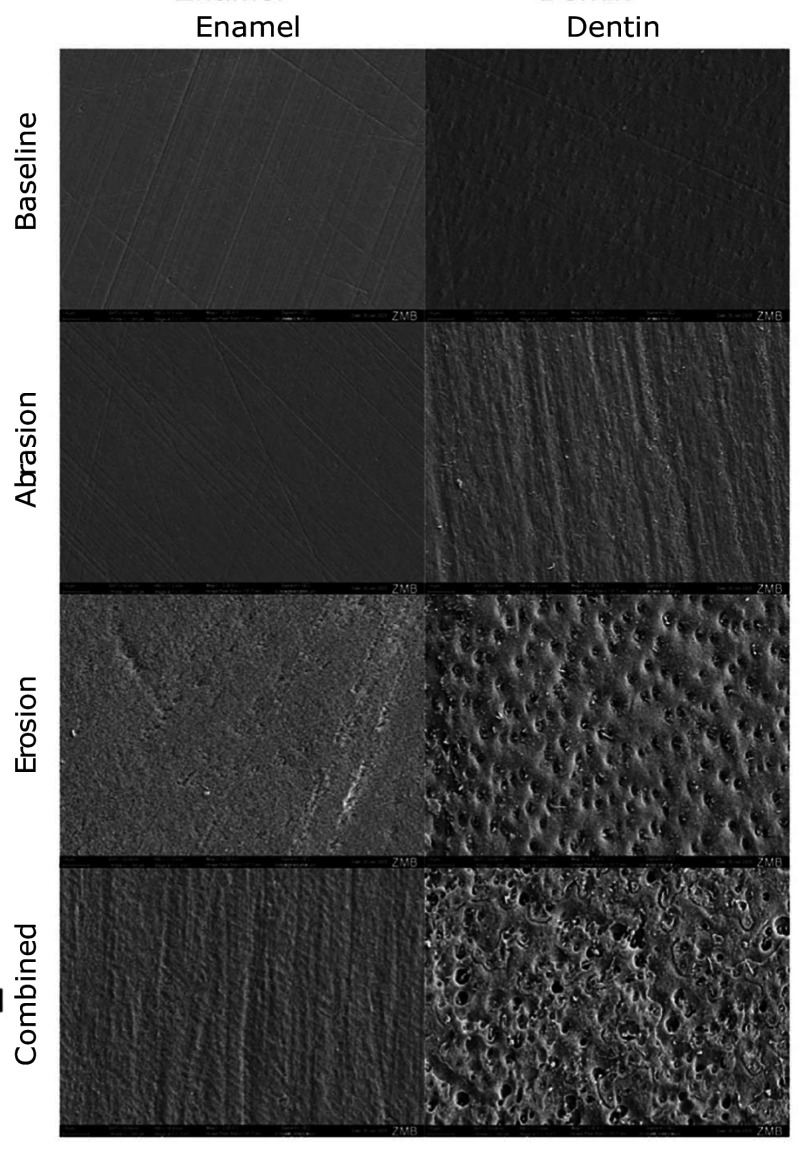
Surface scanning electron microscopy (SEM) images of dentine and enamel at 2,000× magnification at baseline and after 10 days in abrasion, erosion, and combined groups.

The SEM images at 2,000× magnification reveal distinct morphological changes in enamel and dentine surfaces subjected to the different treatment protocols over 10 days (Fig 7).

At baseline, both enamel and dentine surfaces appear relatively smooth, with characteristic parallel striations in enamel and a uniform texture with slight natural porosities in dentine. In the abrasion-only group, enamel maintains its structured appearance. In dentine, abrasion results in more pronounced parallel grooves along the surface in the brushing direction, indicating the mechanical wear through IDBs. In the erosion-only group, enamel shows a rougher surface with an irregular texture compared to baseline. The dentine surface shows an increase in porosity, with visible dentinal tubules, thus indicating demineralisation and loss of structural integrity. In the combined group, enamel appears rough and extensively eroded, with deep parallel grooves in brushing direction and an irregular surface. In dentin, the structural loss is even more severe, with extensive exposure and enlargement of dentinal tubules, indicating pronounced demineralisation and degradation of the dentine matrix.

## DISCUSSION

In this study, bovine teeth were selected for the sample preparation as they were used in various abrasion/erosion studies before.^5,6,27^ Bovine teeth have various advantages compared to human teeth, such as a greater surface area of crown and root, which allows one to gain more samples from one single tooth. This may lead to a higher homogeneity within the samples. Additionally, it is easier to obtain a sufficiently large number of bovine teeth as they are available as a slaughter by-product. Attin et al^8^ discovered that higher enamel loss may occur in bovine teeth compared to human teeth in erosion and erosion-abrasion models, which should be respected when interpreting the results. Bovine dentine can be considered an appropriate alternative to human dentine when abrasion and abrasion-erosion models are applied.^43^ However, wear by erosion alone in human teeth seems to be significantly higher than in bovine mandibular incisors.^43^ Both enamel and dentine samples were polished before the study for an even surface. Although there is no data available on the effect of polishing of bovine enamel and dentine surfaces, an *in-vitro* study of Ganss et al^16^ investigated the difference between polished and natural human enamel and dentine surfaces. In enamel, erosion on polished surfaces was greater than on natural surfaces and in dentin, the erosion was greater on polished radicular dentine than on natural radicular dentine.^16^ For these reasons, the use of bovine teeth as a limitation in this study must be considered.

Another limitation of this *in-vitro* study is the absence of an acquired pellicle, a protein-rich layer that forms as a result of salivary proteins selectively attaching to tooth surfaces *in vivo*.^42^ The obtained pellicle acts as a protective shield, notably reducing the increase in surface roughness and loss of surface microhardness on bovine enamel due to organic acid’s exposure, thereby safeguarding against tooth demineralisation.^42^ Due to its absence, this *in-vitro* model may overestimate the extent of substance loss and surface roughness changes in enamel compared to real-life conditions. Future studies incorporating methods to simulate the protective effects of the acquired pellicle would provide more accurate insights into the in vivo interaction between erosion and abrasion.

To quantify the loss of dental substance in relation to the non-treated reference areas that were taped during erosion cycles, stylus profilometry was used. Profilometry has been used in various studies before to measure surface loss after acid exposure and/or brushing.^11,27,47^ This method yields measurements that are quite precise, with a minimum measurement limit of 0.105 μm and an approximate reproducibility of 40 nm.^2,26^ However, there are some disadvantages pertaining to contact profilometry, such as the possibility of the penetration of the partially eroded enamel or dentine by the stylus scanning the surface, which could lead to damage to enamel and dentine surfaces and an overestimate of surface loss.^33^ Each erosive cycle in this study lasted 2 min and was repeated 10 times per day. The intervals between the cycles, during which the samples were remineralised in artificial saliva, were exactly 20 min long. This protocol is designed to simulate the real-life scenario of repeated reflux with minimal time to remineralise in between. During reflux events, the median gastric pH is 1.4 in healthy individuals and 1.5 in GERD patients not taking proton pump inhibitors (PPI).^31^ The HCl used in this study had a pH of 2.3, estimating the pH in the oral cavity during a reflux event. Numerous studies prior to this one used HCl as the erosive agent as well, with a pH value between 2.2 and 2.6.^3,9,44^ Along with HCl produced by parietal cells, the gastric juice also contains enzymes such as pepsin and trypsin, which were not included in this experiment.^15^ Schlueter et al^34^ proved that dentine samples demineralised with HCl and then treated with a trypsin and a pepsin-trypsin solution for 10 min showed a greater tissue loss than the control group after 6 days. The lack of components of gastric juice, like trypsin and pepsin, during erosive attacks could be a possible limitation of this study. The abrasive challenge with IDBs took place twice a day, once in the morning and once in the evening. Ten brushing cycles, which equals 20 strokes with toothpaste slurry, were chosen as the anticipated number of brushing strokes *in vivo* twice a day.^46^ According to Hamza et al,^19^ the toothpaste used in this experiment (CTO) had a moderate REA of 4 ± 2 and a very high RDA of 100 ± 5.

The results for enamel revealed a clear hierarchy of damage, with the combined erosion-abrasion group experiencing the most substantial substance loss and surface roughness. The null hypothesis that there is no difference in the wear of enamel between the erosion-only, abrasion-only, and combined erosion-abrasion groups has to be rejected. After 5 days, enamel samples in the erosion-only group already showed significant substance loss compared to the abrasion-only group, reflecting the known vulnerability of enamel to acidic environments.^50^ Acidic exposure disrupts the hydroxyapatite crystal lattice, leading to surface demineralisation, which manifests itself in substance loss, an irregular surface, and a reduction in microhardness.^40^ The irregularity of the eroded surface in enamel can be observed in the SEM images (Fig 7). Studies have shown that this softening effect can occur within minutes of acid exposure, leaving enamel more susceptible to mechanical forces.^22^ This was evident in the combined group, where brushing further exacerbated substance loss by removing the softened, partially demineralised enamel. In the SEM images, the vertical brushing direction is visible in the combined group. By day 10, substance loss in the combined group (18.88 ± 0.56 μm) was greater compared to the erosion-only group (14.61 ± 0.81 μm), indicating a synergistic effect of these challenges. As expected, the abrasion-only group exhibited minimal to no substance loss and surface roughness gain on the enamel. A study by Hamza et al^18^ measured 0.1 μm of surface loss after brushing bovine enamel samples for 6 hours with a toothbrush and toothpaste slurry. It can be concluded that daily brushing on enamel surfaces with IDB and a toothpaste with low to moderate REA poses no risk to dental health. Surface roughness measurements provided additional insights into the interaction between erosion and abrasion. The erosion-only group exhibited significant roughness compared to the abrasion-only group, likely due to the irregular demineralisation pattern caused by acid exposure.^49^ It was discovered by Eisenburger et al^14^ and Shellis^37^ that demineralisation at the prism junctions occurs before demineralisation within enamel prisms, thus leaving an irregular surface, which can be seen in the SEM images. However, the combined group showed the highest roughness values, suggesting that brushing disrupted the softened enamel surface unevenly. These findings are consistent with a prior study that demonstrated how mechanical forces can create irregular wear patterns on eroded enamel surfaces.^30^ A solution to this problem of combined erosion-abrasion could be a period of remineralisation after drinking erosive beverages or having reflux and before brushing teeth, as Attin et al^4^ investigated.

The results for dentine demonstrate a clear difference in wear between the three groups, with the abrasion group showing little substance loss, whereas the erosion and combined groups show a more noticeable substance loss. Therefore, the second null hypothesis that there is no difference in the wear of dentine between the erosion-only, abrasion-only and combined erosion-abrasion groups has to be rejected too. After 5 days, abrasion alone caused noticeable substance loss and surface roughness, highlighting the susceptibility of dentine to mechanical wear. Unlike enamel, dentin’s structure – compromising approximately 50 vol% carbonated hydroxyapatite minerals, 30 vol% organic material (primarily collagen), and 20% water – makes it less resistant to mechanical forces.^36^ The change in surface structure by IDBs in the abrasion group can be seen in the SEM images as vertical strokes (Fig 7). Another important factor to consider for the substance loss in the abrasion-only group is the RDA value of the toothpaste used, which was quite high, with a value of 100. In combination with IDBs, this highly abrasive toothpaste seems to have contributed to the dentine wear. As expected, the erosion-only group exhibited a high difference in substance loss and roughness gain after 10 days compared to baseline. In contrast, the difference in substance loss and roughness gain between 5 and 10 days is less. This may be explained by the fact that the erosive demineralisation of dentine leaves an intact surface layer of insoluble collagenous matrix, which cannot be degraded by acid. Matrix metalloproteinases (MMPs) from saliva, which are not present in this *in-vitro* study, or from dentine are activated by a low pH and might be able to degrade the collagenous matrix.^41^ As there is no saliva present in this study, this effect of MMPs on collagenous matrix degradation is rather unlikely to occur in this case. The demineralised layer’s thickness and, consequently, the diffusion distance between the surface and the demineralisation front both grow with erosion, resulting in a gradual decline in the net rate of mineral loss.^20,39^ This may explain the only small difference in substance loss and surface roughness in the erosion-only group in dentine at 5 and 10 days. Surprisingly, the substance loss in dentine was significantly higher in the erosion-only group than in the combined group. Results also revealed that, contrary to expectation, the erosion-only group exhibited statistically significantly more substance loss than the combined group. A study by Wiegand and Schlueter^48^ states that by brushing, the exposed collagen matrix cannot be removed but is rather compressed and could even function as a barrier against further abrasion. Observing the SEM images of dentine (Fig 7), it can be seen that in the erosion-only group, dentine tubuli are clearly visible compared to the combined group, where debris is stuck in between and on the tubuli. Thus, it can be suggested that brushing on eroded dentine may not have a multiplicative effect, contrary to findings that eroded dentine is highly susceptible to toothbrush abrasion.^5,7,12^ From the present study, it can thus be concluded that both brushing with IDBs in combination with toothpaste on eroded dentine and erosion alone cause significant substance loss and should thus be avoided.

The third null hypothesis that the effects of different treatment protocols (abrasion-only, erosion-only, combined erosion-abrasion) on substance loss and roughness gain are independent of the tooth substance (enamel, dentin) can be rejected. Results show that enamel is more susceptible to erosion, whereas dentine is more susceptible to abrasion.

In the abrasion-only group, it can be observed that there is minimal substance loss in dentine compared to no substance loss in enamel. An explanation for this difference may be that the toothpaste used in this study has a relatively low REA compared to the high RDA.^19^ A contributing factor is the softer and more organic-rich structure of dentin, which is more susceptible to tissue removal than the highly mineralised enamel when brushing with IDBs and toothpaste. When exposed to acid without abrasion, both enamel and dentine experienced demineralisation. However, the finding emerged that the erosion-only group exhibited lower substance loss in dentine after 5 and 10 days compared to enamel. This can be explained by the fact that the organic content of dentin, which amounts to approximately 30vol%, is not removed by erosion.^36^ Thus, there is a greater diffusion distance between the surface and the beginning of sound dentin, resulting in a decline in the rate of mineral loss.^20,39^ According to a study by Lussi et al,^29^ this may be partially caused by collagen’s buffering characteristics, which lessen the pH value drop at the demineralisation front. Results also show that in the erosion-only group, there is a higher surface roughness in dentine compared to enamel. This might be explained by the fact that in dentin, a collagen matrix remains, and there is a tubular structure in dentin, and in enamel, the surface occurs more evenly, which can be seen in SEM images of erosion-only groups in dentine and enamel (Fig 7).^41^ The results for combined groups in enamel and dentine were similar to those of erosion-only groups. After 10 days, the surface roughness in enamel was half the roughness in dentin, and the substance loss in enamel was more than double that in dentin. This can be explained by the facts stated above and can be seen in the SEM images (Fig 7). In enamel, a roughened surface with visible patterns of combined mechanical and chemical wear can be seen, whereas in dentine, there is extensive degradation with widened dentinal tubules, showing great roughness and collagen matrix collapse.

## CONCLUSION

Based on this *in-vitro* study and within its limitations, it could be concluded that erosion, the combined effect of both erosion and abrasion, and dentine abrasion significantly contribute to the loss of dental hard tissues. Erosion alone leads to notable structural damage, while abrasion alone results in comparatively lower material loss. The combination of erosion and abrasion has a synergistic effect, causing the highest substance loss. Dentine is more susceptible to these challenges than enamel, highlighting the need for targeted protective measures. A possible solution might be using IDBs before toothbrushing with toothpaste, which creates an abrasive slurry. Although preventive strategies such as the use of anti-erosive toothpastes and soft-bristle toothbrushes can reduce enamel wear, none of those measures completely prevent erosive and abrasive damage. This underlines the importance of comprehensive patient education and the development of additional preventive interventions to mitigate the effects of these challenges on dental hard tissues.

### Acknowledgements

This study was conducted as the Master’s thesis of Lynn Etter and performed at the Center of Dental Medicine, Clinic of Conservative and Preventive Dentistry at the University of Zurich, Switzerland, under the supervision of Prof P.R. Schmidlin.


